# Hamiltonian patterns of age-dependent adaptation to novel environments

**DOI:** 10.1371/journal.pone.0240132

**Published:** 2020-10-02

**Authors:** Grant A. Rutledge, Larry G. Cabral, Brandon J. Kuey, Joshua D. Lee, Laurence D. Mueller, Michael R. Rose

**Affiliations:** Department of Ecology and Evolutionary Biology, School of Biological Sciences, University of California, Irvine, California, United States of America; University of Thessaly School of Agricultural Sciences, GREECE

## Abstract

Our intuitive understanding of adaptation by natural selection is dominated by the power of selection at early ages in large populations. Yet, as the forces of natural selection fall with adult age, we expect adaptation to be attenuated with age. Explicit simulations of age-dependent adaptation suggest that populations adapt to a novel environment quickly at early ages, but only slowly and incompletely at later adult ages. Experimental tests for age-dependent adaptation to a novel diet were performed on populations of *Drosophila melanogaster*. The results support the prediction that populations should perform better on an ancestral, long-abandoned diet, compared to an evolutionarily recent diet, only at later ages. *D*. *melanogaster* populations also perform poorly on a novel diet compared to an evolutionarily recent diet that has been sustained for hundreds of generations, particularly at earlier ages. Additional experiments demonstrate that the timing of the shift to better performance in our populations on the long-abandoned diet is dependent on when the forces of natural selection weaken in the evolutionary history of experimental populations. Taken together, these experimental findings suggest that the forces of natural selection scale the rate of adaptation to novel environments.

## Introduction

Evidence suggests that one cause of declining health in modern human populations is the “mismatch” between past human evolution and the diets we are now consuming [1–4]. Paleo-enthusiasts argue that our recent dependence on foods derived from grass species (e.g. wheat, corn, rice) and milk from ungulates exacerbates such chronic disorders as obesity, type two diabetes, and gut disorders such as Crohn’s disease [1–2]. Some physicians argue that heart disease and dementia are linked to agricultural diets [5–7]. Such authors claim that the 10,000 years or so since the beginning of large-scale agriculture and animal domestication [8] was too little time for evolution to adapt our species to the agricultural diet and lifestyle. Therefore, they suggest, humans can best optimize metabolism and physiology when they consume a diet more like that of our ancestors before the advent of agriculture. Moreover, human populations that have recently adopted the agricultural diet exhibit dramatic declines in health [9], which makes it plausible that adopting a Paleolithic, hunter-gatherer diet would alleviate many chronic disorders in newly agricultural groups. But it does not demonstrate the validity of the view that all contemporary human populations would similarly benefit from “Paleo” diets and lifestyles, a view that we will refer to here as the “*Paleo hypothesis*.”

On the other hand, Zuk [10] has argued that our evolution has featured enough time to adapt humans to agriculture among those populations with long-agricultural ancestry. Lindeberg [4] provides data on maintenance of lactase function with age as well as the incidence of chronic diabetes on present-day diets as a function of agricultural history (see Fig 4.20 in [4]). Specifically, these data show that the maintenance of lactase function depends on the historical duration of dairy consumption, across human populations. Furthermore, lab experiments demonstrate that when environmental conditions change, it takes surprisingly few generations for populations to adapt to new conditions [11]. This speed and effectiveness of experimental evolution suggests that humans could already be well adapted to agricultural diets and activity levels, and that there are no health benefits to be achieved from reverting to Paleolithic diets for most inhabitants of industrialized countries. We will refer to this as the “*anti-Paleo hypothesis*.”

Recently, we have developed an evolutionary hypothesis for the effects of diets from different phases of a species’ evolutionary history based on Hamilton’s [12] forces of natural selection. Hamilton’s forces of natural selection provide scaling factors for the effect of selection on age-specific components of life history, scaling factors that usually decline with increasing age. Theoretical work by Brian Charlesworth and others (e.g. [13, 14]), as well as abundant data from specific fields like quantitative genetics and experimental evolution (vid. [15, 16]), have shown how seminal Hamilton’s forces of natural selection have been for explaining the presence, rate, and now even the cessation of aging [17]. Theoretically, selection for adaptation to novel environments is expected to be strongest at younger ages, but then fall toward low intensities during later adult life. This suggests the hypothesis that when adaptation to a novel environment is at least somewhat age-specific, then natural selection will produce more rapid adaptation at early ages compared to later ages, due to the weaker forces of natural selection at later ages [14].

A corollary of this general hypothesis for humans specifically is that, when humans with long-agricultural ancestry are young, they are well adapted to agricultural diets and activity patterns. But at later adult ages, with enough age-specificity of the alleles that established our adaptation to agriculture, humans may lose their ability to digest agricultural foods or to cope with the patterns of physical labor characteristic of agricultural life. Thus, our evolutionary hypothesis of age-dependent adaptation suggests that people with long-standing agricultural ancestry might benefit from adopting a Paleolithic diet only at later adult ages. We call this the “*Hamiltonian hypothesis”*. An explicit model for transient age-dependent adaptation supports the intuitive expectation that populations adapt quicker to a novel environment at early ages and slower at later ages (Phung et al. in prep.). Here we will test the Hamiltonian hypothesis using an experimental paradigm featuring dietary transition.

Since the summer of 1981, the long-standing laboratory *Drosophila melanogaster* populations used in this study have been maintained exclusively on medium that contains banana and high-sugar syrups as the chief substrates [16]. The wild *D*. *melanogaster* population from which these laboratory populations were derived is that of Northeastern United States; the local agricultural setting is one that has featured apples as the chief cultivated fruit for centuries [18]. *D*. *melanogaster* is native to equatorial Africa and was likely introduced to North America from Europe a few centuries ago [19]. No banana cultivation occurred over the three centuries or so that wild *D*. *melanogaster* lived in this region, prior to our founding of laboratory populations from them. As a result, we have a model system which features one long-standing dietary regime, dominated by apple rot, being replaced with another long-standing dietary regime, banana with live yeast.

To further study patterns of adaptation to novel environments, we can also impose a novel dietary regime on these flies. The entirely novel dietary regime we use here features oranges as the chief substrate. No orange cultivation occurred in the region inhabited by the wild *D*. *melanogaster* we sampled.

If the age-dependent hypothesis is correct, then we expect our lab populations to fare as well or better on banana as on apple at early ages. However, at much later ages, we predict that these populations should perform better on apple medium, when the forces of natural selection have weakened enough to forestall sufficient adaptation to the banana food provided more recently in their evolutionary history. Furthermore, we expect that flies on banana should outperform flies on an evolutionarily novel diet at early ages. But at later ages, we expect the functioning of flies given entirely novel diets to converge on that of flies fed banana, because of a lack of adaptation at later ages to the banana diet.

**Fig 1:**
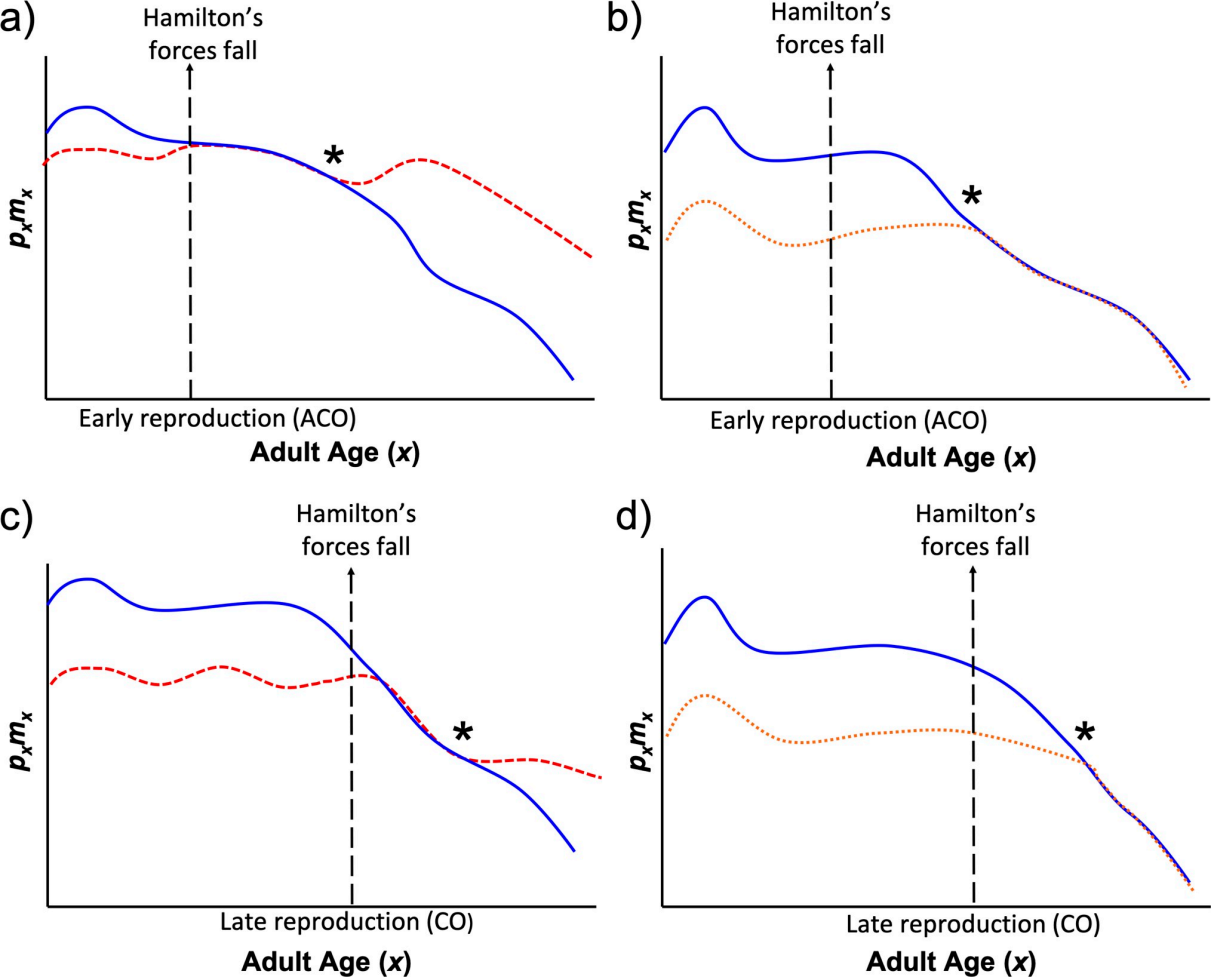
Hypothetical *p*_*x*_*m*_*x*_ curves of flies from two different demographics exposed to various diets. *p*_*x*_*m*_*x*_ is age-specific survival probability (*p*_*x*_) multiplied by age-specific female fecundity (*m*_*x*_). Dashed red trend lines represent *p*_*x*_*m*_*x*_ on a long-abandoned diet, solid blue on an evolutionarily recent diet, and dotted orange on an entirely novel diet. (a) Flies evolving under an early reproduction regime should fare as well or better on their evolutionarily recent diet as on a past ancestral diet at early ages. But at later ages, we predict that individuals should fare better on a long-abandoned diet when the forces of natural selection have weakened enough to prevent sufficient adaptation to the food imposed in recent evolutionary time. (b) Flies should perform significantly better on an evolutionarily recent diet as opposed to an entirely novel diet, particularly at early adult ages. (c) If reproduction is pushed to later ages, we would expect a longer period of sustained function on the evolutionarily recent diet and a switch to superior function with the long-abandoned diet to occur at later ages or not at all as indicated by the asterisk. (d) Our theory also implies a longer period of sustained function on the recent diet compared to an entirely novel diet. Convergence in these trends would be expected at even later ages as indicated by the asterisk. This last prediction was not tested in our experiments.

Moreover, we predict that the timing of this “switch” to better performance on the apple diet should depend on the life cycle imposed by the culture regime; that is, it should be dependent on when Hamiltonian forces weaken. In other words, if the reproductive window of our populations is pushed to later ages, we predict a longer period of sustained function on the banana diet, with the benefits of a switch to the apple diet occurring at later ages or perhaps not at all. We test this hypothesis using sets of populations that have long had two different, age-specific, reproductive windows. Our qualitative predictions for the patterns of age-specific adaptation to this assortment of diets are shown in Fig 1.

## Materials and methods

### Populations

We used large, outbred populations of *Drosophila melanogaster* selected for two different patterns of age-specific reproduction. Five “ACO” replicates (ACO_1-5_) have been adapted to banana-molasses food for ~1000 generations and have a 10-day life cycle. Five “CO” replicates (CO_1-5_) have been adapted to banana-molasses food for ~500 generations and have a 28-day life cycle (S1 Fig). A more detailed and up-to-date description of the history and culture methods for the ACO and CO populations can be found in Burke et al. [20].

### Experimental design

A total of three diet-manipulation experiments were performed in the lab: ACO replicate populations 1–5 were assayed on the apple and banana diets. ACO replicate populations 1–3 were assayed on the banana and orange diets (ACO 4 and 5 were not used due to resource limitations at the time). CO replicate populations 1–5 were assayed on the banana and apple diets.

### Food preparation

The evolutionarily recent banana-molasses food given to fly cohorts is composed of the following ingredients per 1L distilled H_2_0: 13.5g Apex^®^ Drosophila agar type II, 121g peeled, ripe organic banana, 10.8mL light Karo^®^ corn syrup, 10.8mL dark Karo^®^ corn syrup, 16.1mL Eden^®^organic barley malt syrup, 32.3g Red Star^®^ active dry yeast, 2.1g Sigma-Aldrich^®^ Methyl 4-hydroxybenzoate (anti-fungal), and 42.5 mL 95% ethanol. The novel orange food was prepared identically to the banana-molasses food except peeled banana was replaced with juice and pulp from peeled oranges. The long-ancestral apple food is prepared in the same manner as the banana food, except the diet lacks the barley malt and corn syrups, and we substitute organic peeled applesauce for the peeled banana. This is our best emulation of the ancestral diet of our lab flies. Basic nutritional facets of each diet are shown in S1 Table. Our experimental cohorts are exposed to their respective diets throughout their developmental (larval) stage and adulthood.

### Mortality and fecundity assays

All populations were reared in polystyrene vials with the respective diet and given 9 (ACO) and 14 (CO) days to develop. Adult flies were transferred into 6L acrylic glass cages on the 9^th^ (ACO) and 14^th^ (CO) day using CO_2_ anesthesia and given fresh food every day with 1mL supplemented yeast (98mL distilled water, 2g active dry yeast, and 2mL 1% acetic acid). Individual mortality was assessed every 24 hours, the flies were sexed at death, and the observed cohort size was calculated from the complete recorded deaths. During the assay, flies were transferred to clean cages once a week using CO_2_ anesthesia to prevent the buildup of feces, which made assessing mortality difficult and may have subjected flies to higher levels of ammonia. At the start of the assay, cohorts were assayed in 6L cages at ~1000 flies per cage. Flies were transferred to 3L cages at 50% starting cohort size to control for density effects. Age-specific fecundity was also assessed every 24-hours, being estimated from the number of eggs laid by females on the culture medium plates placed in each mortality assay cage, divided by the number of females still alive. Media plates were washed onto filter paper with the lab’s fecundity funnel system and then scanned for counting at a later time [20]. Egg counting was performed using ImageJ (imagej.nih.gov/ij/index.html), a National Institute of Health validated image-processing program [21].

### Statistical analysis

#### *p*_*x*_*m*_*x*_ analysis for ACO experiments

The age-specific survival probability (*p*_*x*_) is the probability of a female surviving to age *x*, given that she survived to the start of the age-interval. It is calculated using the following equation:

$$p_{x}=1-\left(\frac{d_{x}}{n_{x}}\right)$$ (1)

where *d_x_* is the number of females that die at age *x*, and *n_x_* is the number of females that were alive at the start of age *x*. Age-specific fecundity (*m*_*x*_) is the average number of eggs laid per surviving female at age *x*. The product of these two variables gives an overall estimate of how cohorts of females are functioning at each age. In our experiments, the unit interval for *x* is a single day.

For all three diets (banana, apple, and orange), *p*_*x*_*m*_*x*_ remained roughly steady until a “breakday” when we see a linear decline in this parameter until day 39 (S2 Fig). The model we fit to this data was a three-parameter two-stage linear model with the following relationship between age (*x*) and *p*_*x*_*m*_*x*_:

$$p_{x}m_{x}=\left\{ \begin{array}{c} a_{0},if\;x\leq a_{2}\\ a_{0}+a_{1}(x-a_{2}),if\;x>a_{2,} \end{array}\right.$$ (2)

where *a*_*0*_ is the *y*-intercept of the first stage, *a*_*1*_ is the slope of the second stage, and *a*_*2*_ is the breakday. The model was fitted using all the *p*_*x*_*m*_*x*_ data at each age starting on the first day of the assay (day 10 from egg). This model was fit to the data using a nonlinear least-squares function in the R-project for statistical computing (r-project.org; version 3.3.3) [22]. We wrote a self-starting R-function for the two-stage linear model that provided initial estimates for the parameter values as well as the predicted *p*_*x*_*m*_*x*_ from Eq (2). A significance value of 0.05 (α) was used to test the null hypothesis that the slopes or *y*-intercepts of the two linear regressions for each diet for each regression analysis were not different.

#### *p*_*x*_*m*_*x*_ analysis for CO experiment

CO data did not follow the same trend as the ACO data and was thus analyzed using a different statistical approach. In this experiment, we tested for differences in *p*_*x*_*m*_*x*_ in seven five-day age-classes in CO populations exposed to the apple and banana diets. The observations consisted of *p*_*x*_*m*_*x*_ at an age (*x*) within an age-interval-*k* (*k* = 1, 2,…,7). Within each interval, *p*_*x*_*m*_*x*_ was modeled by a straight-line allowing diet-*j* (*j* = 1 for banana, or *j* = 2 for apple) to affect the intercept, but not the slope of the line. The slope could vary between intervals. Populations-*i* (*i* = 1, 2…,10) contributed random variation to these measures. With the notation above, the *p*_*x*_*m*_*x*_ at age (*x*), interval (*k*), diet (*j*), and population (*i*) is *y*_*ijkx*_ and can be described by,

$$y_{ijkx}=\alpha +\beta_{k}+\delta_{j}\gamma_{j}+(\omega +\pi_{k}\delta_{k})x+\delta_{k}\delta_{j}\mu_{jk}+c_{i}+E_{ijkx},$$ (3)

where *δ*_s_ = 0 if *s* = 1 and 1 otherwise, and *c_i_* and $E_{ijkx}$ are independent standard normal random variables with variance $\sigma_{c}^{2}$ and $\sigma_{E}^{2}$, respectively. The effects of diet on the intercept are assessed by considering the magnitude and variance of both *γ_j_* and *μ_jk_* [20]. Statistical computing was completed in R (r-project.org; version 3.3.3) [22].

#### *p*_*x*_ survivorship analysis

For each combination of diet*sex, three-day survivorship intervals were computed in R (r-project.org; version 3.3.3) [22]. For each interval a new categorical variable was then created, defining the status of each one of the flies (0 = dead or 1 = alive). The counts of each interval were used in a chi-square test to compare diets (ACO banana vs. orange, ACO apple vs. banana, or CO apple vs. banana). A Bonferroni correction was applied to correct for the multiple age-classes per comparison. A *p*-value of less than 0.05 (α) was considered statistically significant.

## Results

### ACO apple and banana diet results

**Fig 2:**
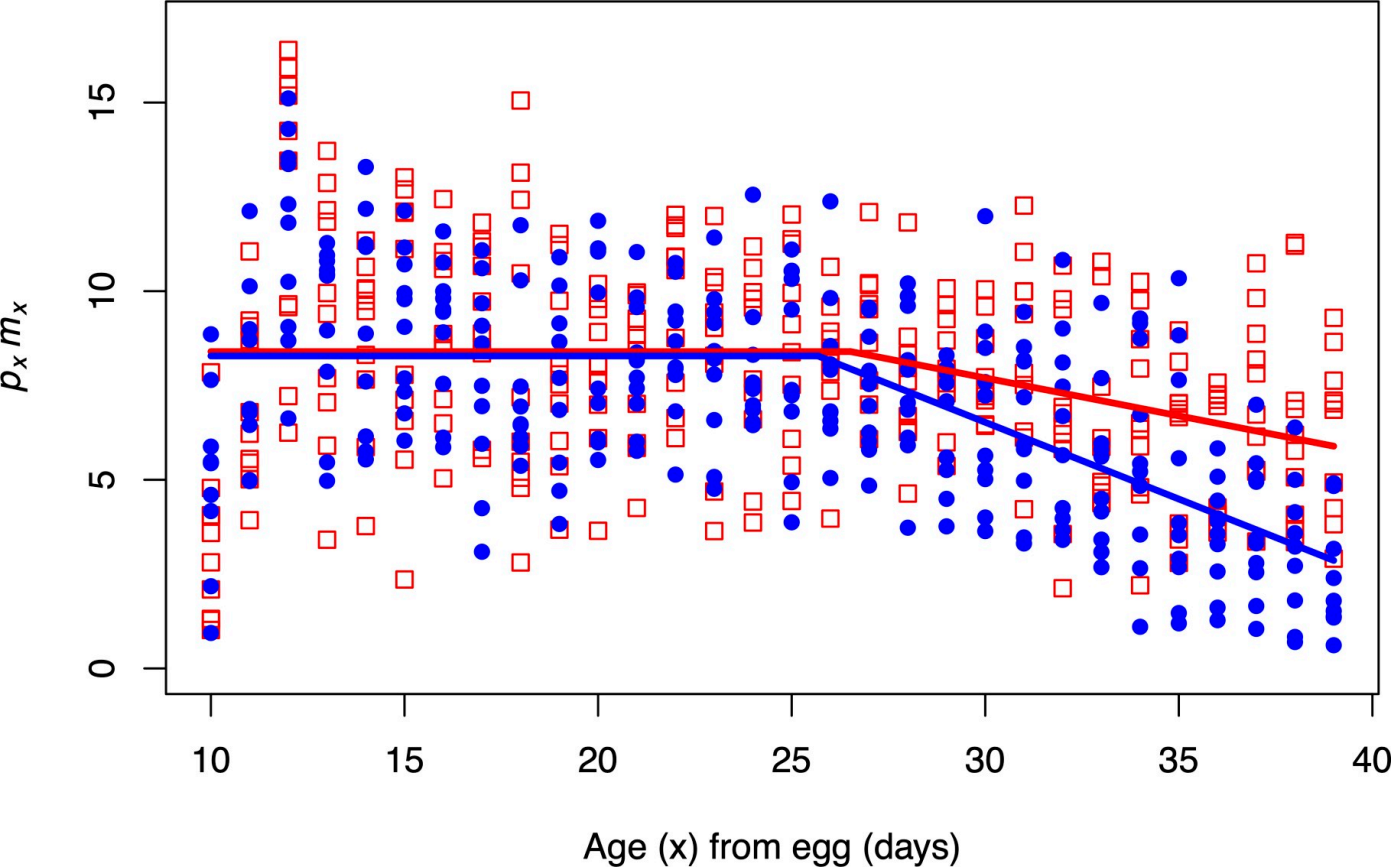
*p*_*x*_*m*_*x*_ results for the five ACO populations on the apple and banana diets. Closed blue circles and solid blue lines represent data and regressions from flies fed the banana diet. Open red squares and dotted red lines represent data and regressions from flies fed the apple diet. A significant difference between diets in the intercept from the first stage (*a*_0_) was not observed. There was no significant difference in the breakday (*a*_2_) between diets. Lastly, the second-stage slope (*a*_1_) was significantly more negative for the banana diet.

For *p*_*x*_*m*_*x*_, the *y*-intercepts of the first stage regression (*a*_0_) for each diet were not statistically different (*p* = 0.763, Table 1). Flies performed similarly on the banana and apple diets at ages up to the breakday (~26 days from egg) (Fig 2). The breakdays (*a*_2_) for each diet were also not statistically different (*p* = 0.775, Table 1). The second stage slope (a_1_) for the banana diet was significantly more negative than for the apple diet (*p* = 0.00920, Table 1). In other words, *p*_*x*_*m*_*x*_ declined faster on the banana diet after the breakday, relative to the apple diet (Fig 2). Recall that our prediction was a less rapid decline in *p*_*x*_*m*_*x*_ at later ages among flies given our crude evocation of their ancestral diet, compared to the decline expected with their recent banana diet. We found that the regression of *p*_*x*_*m*_*x*_ on age with the apple diet to be statistically less negative at later ages, compared to the regression of *p*_*x*_*m*_*x*_ on age with the banana diet. We observed no statistical difference for *p*_*x*_*m*_*x*_ between the two diets at earlier ages.

**Table 1:** Parameter estimates from the two-stage linear model fitted to *p*_*x*_*m*_*x*_ data from the ACO populations on the banana and apple diets.

	First stage	Second stage	breakday
	*y*-int (*a*_0_)	Slope (*a*_1_)	(*a*_2_)
apple	8.398	-0.201	26.51
banana	8.283	-0.407	25.68
*p*-value	0.763	**0.0092**	0.775

*p*-values less than 0.05 are bolded.

When analyzing age-specific survivorship (*p*_*x*_), several intervals were significantly higher on the banana diet compared to the apple diet (S4 Fig). Particularly, male and female flies recently eclosed from pupae (day 10–12 from egg) showed higher mortality rates on the apple diet, suggesting that performance was lower on the apple diet compared to the banana diet at juvenile ages prior to adult (S3 Fig). The survivorship and mortality curves for these diets are shown in S4 and S5 Figs, respectively.

### ACO orange and banana diet results

**Fig 3:**
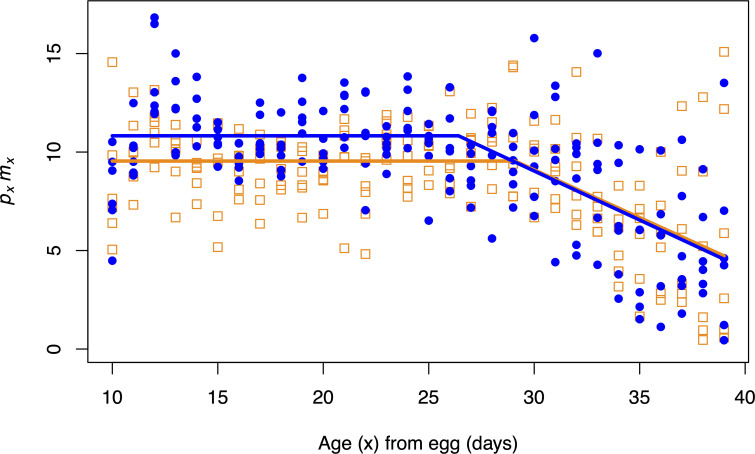
*p*_*x*_*m*_*x*_ results for the three ACO populations on the banana and orange diets. Closed blue circles and solid blue lines represent data and regressions from the banana diet. Open orange squares and dotted orange lines represent data and regressions from the orange diet. A significant difference between diets in the intercept from the first stage (*a*_0_) was observed with flies on the orange diet not performing as well compared to the banana diet. In addition, there was no difference in the breakday (*a*_2_) between diets. Lastly, the second-stage slope (*a*_1_) was not significantly different between the two diets.

For *p*_*x*_*m*_*x*_, the *y*-intercepts of the first stage regressions (*a*_0_) for each diet were statistically different (*p* = 0.043, Table 2). Flies perform better on the banana diet compared to the orange diet at ages up to the breakday (~28 days from egg, Fig 3). The breakdays (*a*_2_) for each diet were not statistically different (*p* = 0.123, Table 2). The second stage slope (a_1_) for the banana diet and the orange diet were also not significantly different (*p* = 0.922, Table 2). *p*_*x*_*m*_*x*_ declined at a similar rate on the banana and orange diet after the breakday (Fig 3). Our prediction was a lower regression for the orange diet at earlier ages compared to the banana diet. We found that the regression of *p*_*x*_*m*_*x*_ on age with the orange diet to be statistically lower at ages up to the breakday, compared to the regression of *p*_*x*_*m*_*x*_ on age with the banana diet. We observed no statistical difference for *p*_*x*_*m*_*x*_ between the two diets at later ages. Performance after the breakday was similar between the two diets. Results from the survivorship analysis for the banana and orange diets are shown in S6 Fig. The survivorship and mortality curves for these diets are shown in S7 and S8 Figs, respectively.

**Table 2:** Parameter estimates from the two-stage linear model fitted to *p*_*x*_*m*_*x*_ data from the ACO populations treated with the banana and orange diets.

	First stage	Second stage	breakday
	*y*-int (*a*_0_)	Slope (*a*_1_)	(*a*_2_)
orange	9.543	-0.486	29.091
banana	10.826	-0.498	26.412
*p*-value	**0.043**	0.922	0.123

*p*-values less than 0.05 are bolded.

### CO apple and banana results

**Fig 4:**
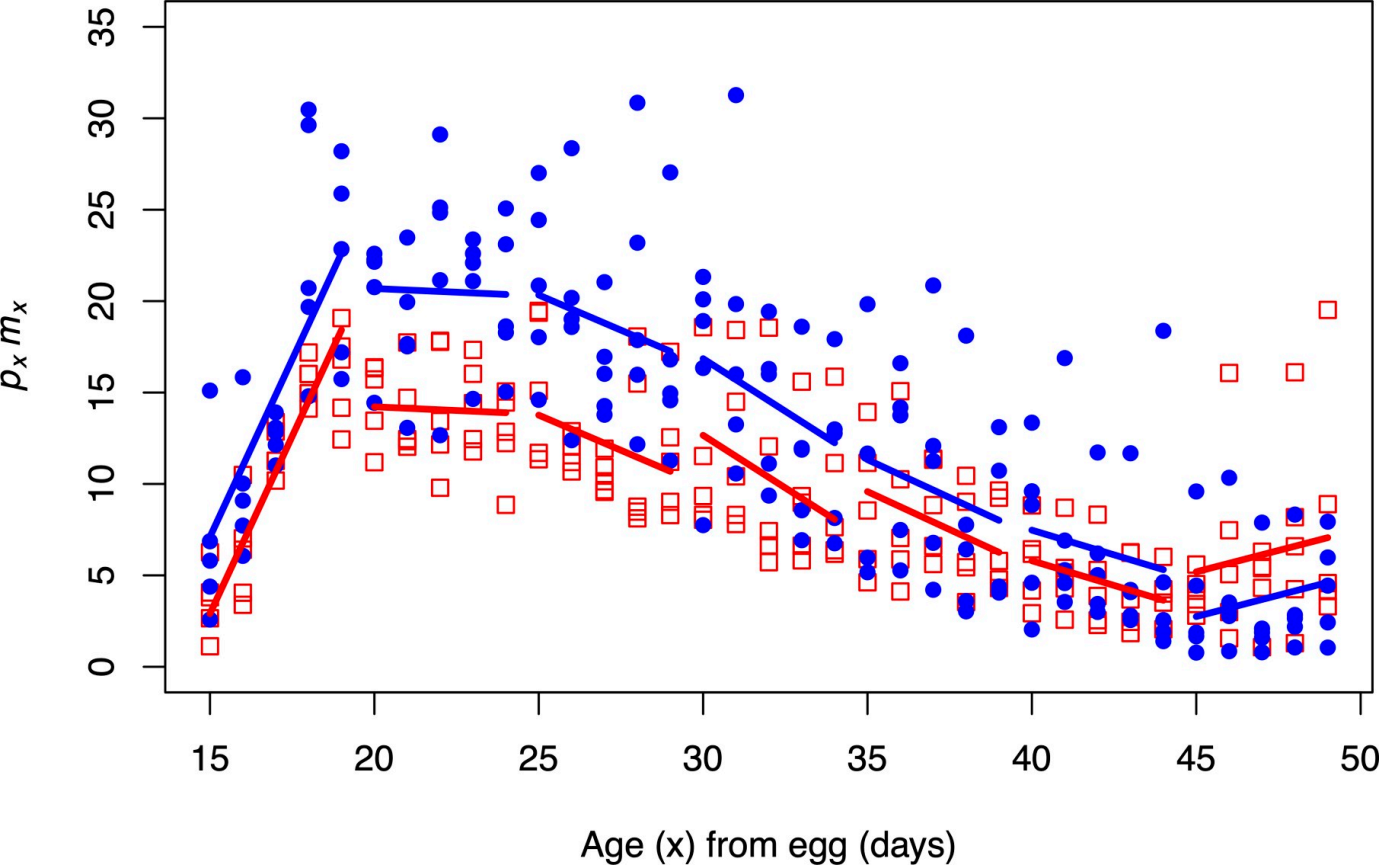
*p*_*x*_*m*_*x*_ results in five CO populations exposed to the apple and banana diets. Closed blue circles and solid blue lines represent data and regressions from the banana diet. Open red squares and dotted red lines represent data and regressions from the apple diet. Flies performed significantly better on the banana diet for the second and third age-classes. All other age-classes are not significantly different with respect to their performance on the two diets.

For the first age-interval (days 15–19 from egg), we did not observe a significant difference between the two diets (*p* = 0.0633, Table 3, Fig 4). For the following two age-intervals (days 20–24 & 25–29 from egg), we observed significantly higher *p*_*x*_*m*_*x*_ intercepts on the banana diet compared to the apple diet (*p* < 0.05, Table 3, Fig 4), followed by convergence in the last four age-intervals (*p* > 0.05, Table 3, Fig 4). For the last age-interval, we see a switch to higher performance on the apple diet; however, this was not statistically significant. The results of this experiment fit the prediction of sustained CO function on banana further into adult age, compared to the ACO flies. Furthermore, we obtained the expected convergence in *p*_*x*_*m*_*x*_ trends at later age classes. We observed higher *p*_*x*_*m*_*x*_ on the apple diet for the last interval, but this difference was not significant. Age-specific survival curves are shown in S9 Fig. Survivorship and mortality curves are shown in S10 and S11 Figs, respectively.

**Table 3:** Estimates of *p*_*x*_*m*_*x*_ intercepts for each age-class (days from egg) in the CO populations on the banana and apple diets and *p*-value of their statistical comparison.

Age-class	Age	*p*_*x*_*m*_*x*_ apple-banana	*p*-value
15–19	3.88	-4.20	0.0633
20–24	-0.08	-6.90	**0.0106**
25–29	-0.77	-6.58	**0.0098**
30–34	-1.15	-4.19	0.0638
35–39	-0.83	-1.75	0.394
40–44	-0.54	-1.68	0.4152
45–49	0.47	2.45	0.2455

*p*_*x*_*m*_*x*_ estimates represent the *y*-intercepts of a linear regression through all replicates of five-day intervals. *p*-values less than 0.05 are bolded.

## Discussion

Our experimental findings provide support for the Hamiltonian hypothesis of age-dependent adaptation and suggest that the forces of natural selection scale the rate of adaptation to novel environments. The results of the diet manipulation experiments using the ACO populations indicate that adaptation appears to conform to Hamiltonian predictions: younger adult flies fare well on their evolutionarily recent banana diet, while older adult flies fare better on medium that is relatively more like their evolutionarily ancestral apple diet. Notably, this occurs despite their lack of exposure to this long-abandoned ancestral diet for more than 1,000 generations of laboratory evolution. Survivorship analysis in these populations indicates that there may be a decrease in functional health at early ages on the apple diet compared to the banana diet (S3 and S5 Figs), however the *p*_*x*_*m*_*x*_ analysis did not detect a difference. The results from the CO experiment provide more evidence for the Hamiltonian hypothesis. These late-cultured flies performed significantly better on the banana diet at ages prior to the later reproductive window and the fall in Hamilton’s forces. These flies performed better on the apple diet in the last interval, though this difference was not statistically significant.

The findings displayed in Figs 2–4 were obtained from experiments that studied *D*. *melanogaster* populations that had an inadvertent dietary transition during their evolution. In other words, we did not directly monitor forward selection to a novel diet. However, by performing diet manipulation experiments on such populations with well-characterized dietary histories, we still can test whether populations are conforming to the Hamiltonian hypothesis of age-dependent adaptation, rather than the patterns expected from both Paleo and anti-Paleo hypotheses, neither of which consider age-specificity.

In these experiments, we have shown that age-specific adaptation to a novel environment proceeds at different rates for early-life phenotypes vs. late-life phenotypes, in large-scale experiments. The ages at which we might expect to see a rapid transition from a maladaptive phenotype to a well-adapted phenotype depend on several factors: the time since moving to the new environment; the severity of selection acting on new genetic variants; the magnitude of the phenotypic effects among new mutants; and the effective population size. Since the present findings should apply to any population that has undergone a recent dietary transition, there is the possibility of testing our general Hamiltonian hypothesis further using populations in which there has been a sustained change in diet like that of our *D*. *melanogaster* populations. Additional theoretical and experimental work on the Hamiltonian hypothesis is an obvious next step for this research.

## Supporting information

S1 Fig Schematic of the lifecycle of the populations used in our experiments. ACO and CO populations are reared in vials for the first 9 and 14 days, respectively. On the 9^th^ and 14^th^ day, adults are transferred to cages and maintained until their respective reproductive windows. Eggs laid during the reproductive window are used to start the next generation.
(TIF)

S2 Fig The model used to fit the ACO data from our diet manipulation experiments. From the start of the assay (day 10) until the breakday (*a*_*2*_), *p*_*x*_*m*_*x*_ at age *x* is described by the horizontal line *p*_*x*_*m*_*x*_ = *a*_*0*_. After *a*_*2*_, *p*_*x*_*m*_*x*_ begins to decline and is described by the line *p*_*x*_*m*_*x*_ = *a*_*0*_ *+ a*_*1*_(*x-a*_*2*_).
(TIF)

S3 Fig ACO conditional survival probability (*p*_*x*_) over adult age from egg (*x*) for the apple and banana diets. (a) males and (b) females. Points represent *p*_*x*_ pooled across replicates and pooled across three days. *denotes significance for the particular interval between shown diets (*p*<0.05). The color of the asterisk signifies which diet has higher survivorship for that interval.
(TIF)

S4 Fig ACO survivorship for the banana and apple diets. (a) sexes pooled. (b) male flies. (c) female flies. Data are pooled across replicates.
(TIF)

S5 Fig Natural log transformed age-dependent mortality for the ACO apple and banana diets. (a) sexes pooled. (b) male flies. (c) female flies. Data are pooled across replicates.
(TIF)

S6 Fig ACO conditional survival probability (*p*_*x*_) over adult age from egg (*x*) for the banana and orange diets. (a) males and (b) females. Points represent *p*_*x*_ pooled across replicates and pooled across three days. *denotes significance for the particular interval between shown diets (*p*<0.05). The color of the asterisk signifies which diet has higher survivorship for that interval.
(TIF)

S7 Fig ACO survivorship for the banana and orange diets. (a) sexes pooled. (b) male flies. (c) female flies. Data is pooled across replicates.
(TIF)

S8 Fig Natural log transformed age-dependent mortality for the ACO banana and orange diets. (a) sexes pooled. (b) male flies. (c) female flies. Data are pooled across replicates.
(TIF)

S9 Fig CO conditional survival probability (*p*_*x*_) over adult age from egg (*x*) for the banana and apple diets. (a) males and (b) females. Points represent *p*_*x*_ pooled across replicates and pooled across three days. *denotes significance for the particular interval between shown diets (*p*<0.05). The color of the asterisk signifies which diet has higher survivorship for that interval.
(TIF)

S10 Fig CO survivorship for the banana and apple diets. (a) sexes pooled. (b) male flies. (c) female flies. Data are pooled across replicates.
(TIF)

S11 Fig Natural log transformed age-dependent mortality for the CO banana and apple diets. (a) sexes pooled. (b) male flies. (c) female flies. Data are pooled across replicates.
(TIF)

S1 Table Basic nutritional facets for the three different diets used in the diet manipulation experiments. (DOCX)
