# Supplementary figures and images for: Hamiltonian patterns of age-dependent adaptation to novel environments
Source: PLoS One. 2020 Oct 2;15(10):e0240132. doi: 10.1371/journal.pone.0240132 (PMC7531798; doi:10.1371/journal.pone.0240132)

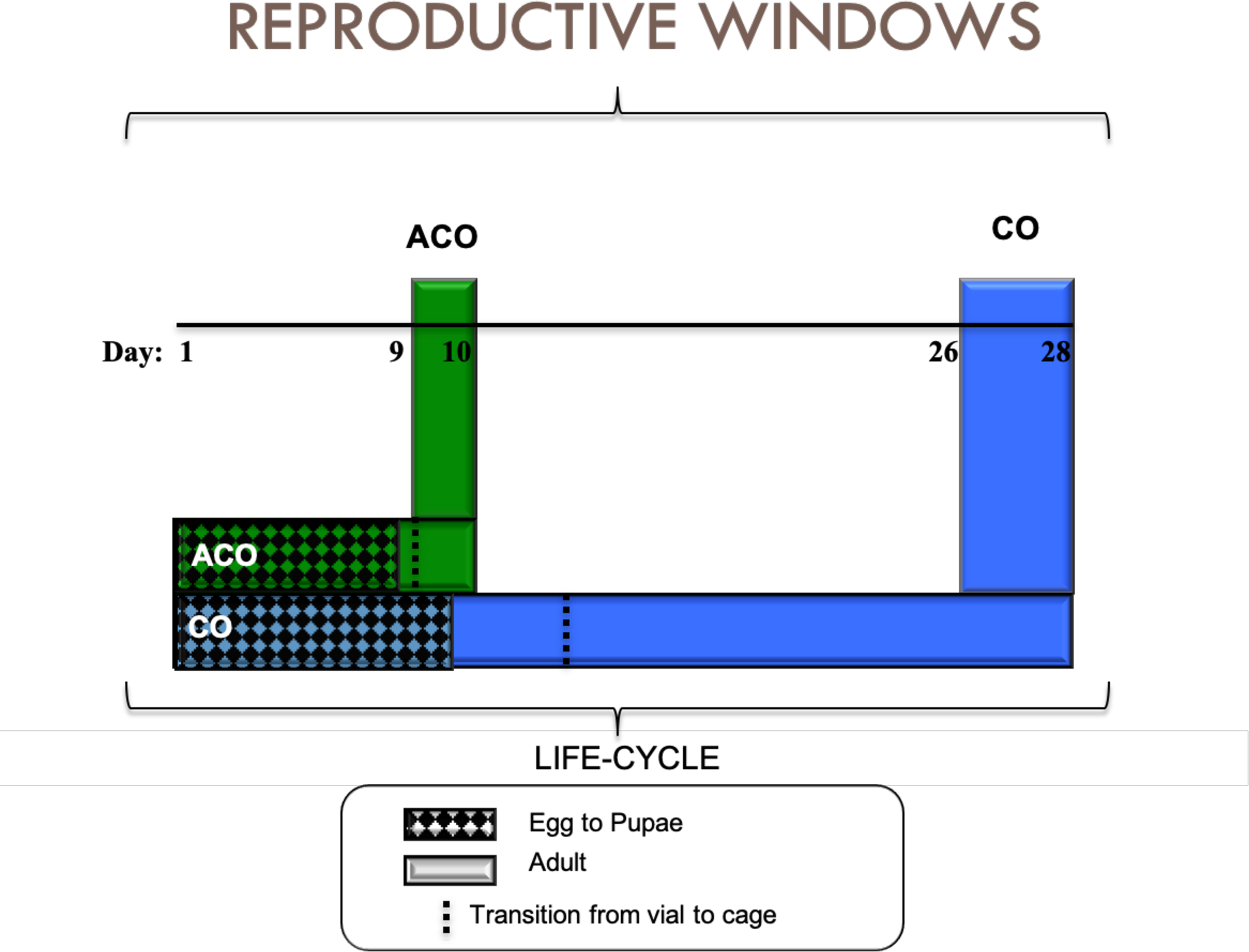

Supplement: S1 Fig — ACO and CO populations are reared in vials for the first 9 and 14 days, respectively. On the 9th and 14th day, adults are transferred to cages and maintained until their respective reproductive windows. Eggs laid during the reproductive window are used to start the next generation. (TIF) [file pone.0240132.s001.tif]

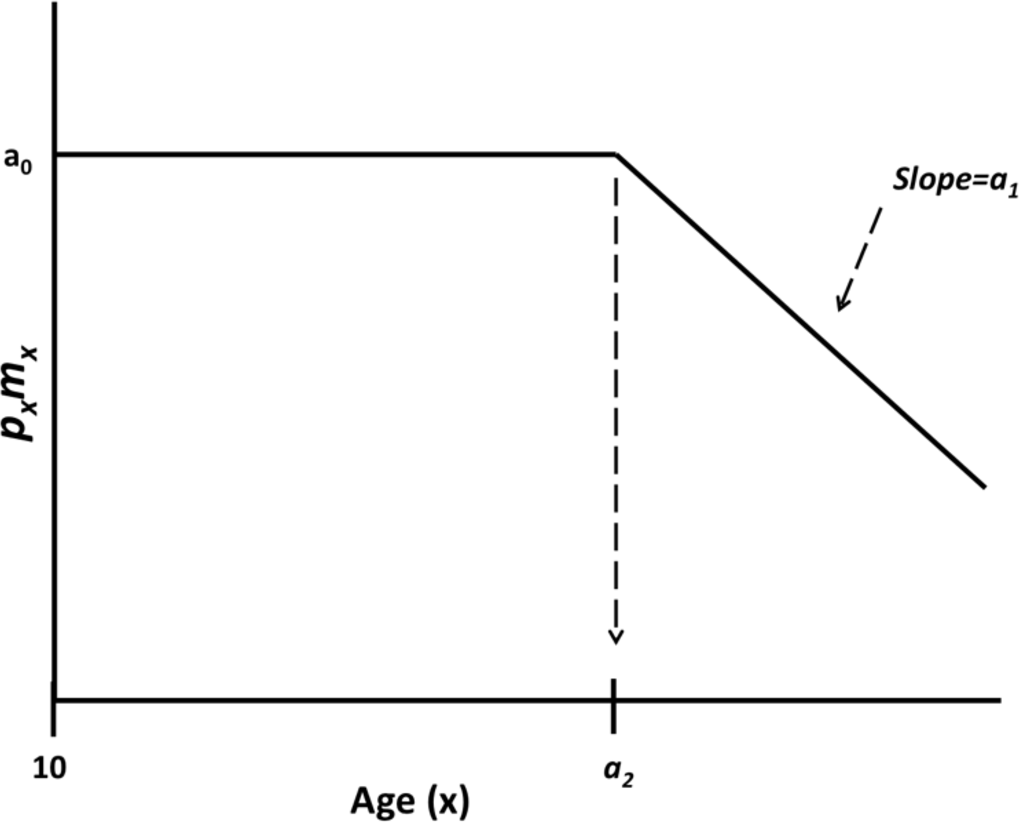

Supplement: S2 Fig — From the start of the assay (day 10) until the breakday (a2), pxmx at age x is described by the horizontal line pxmx = a0. After a2, pxmx begins to decline and is described by the line pxmx = a0 + a1(x-a2). (TIF) [file pone.0240132.s002.tif]

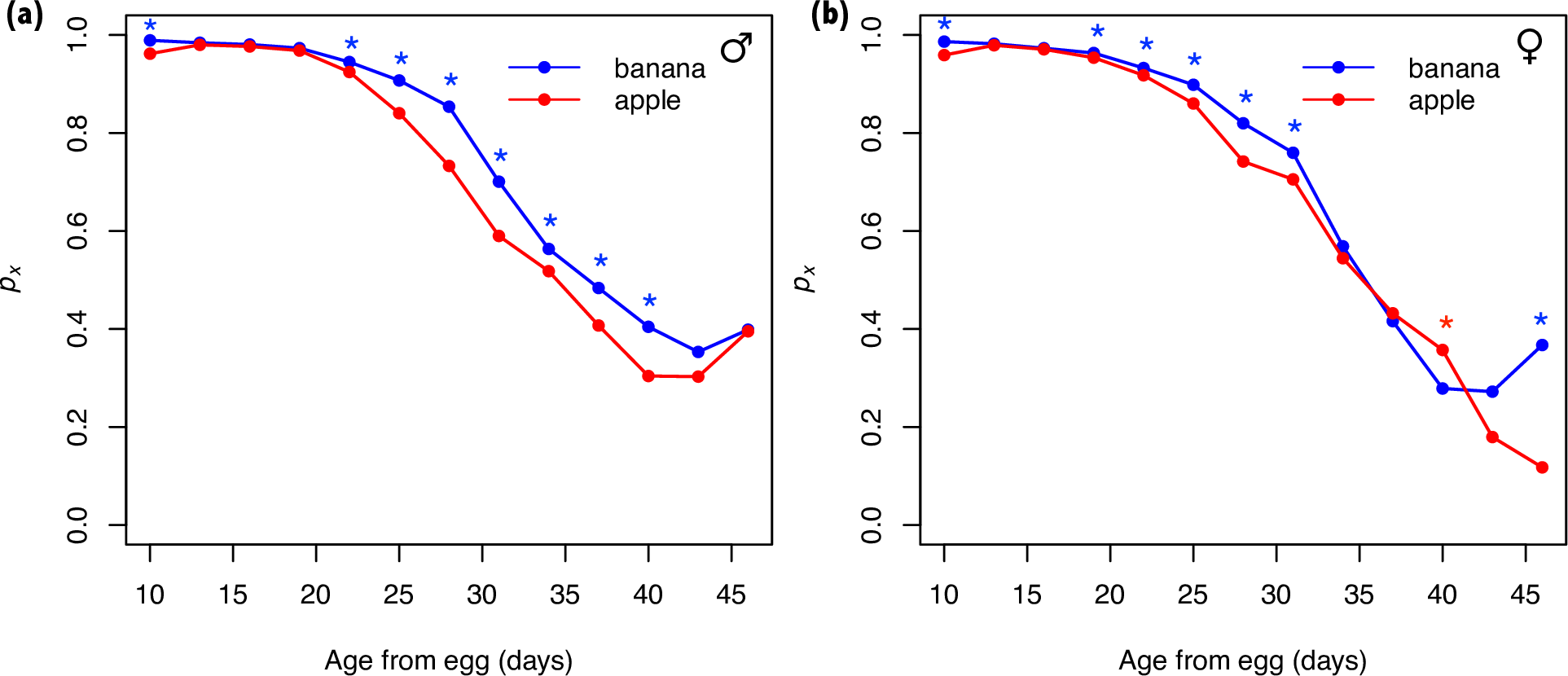

Supplement: S3 Fig — (a) males and (b) females. Points represent px pooled across replicates and pooled across three days. *denotes significance for the particular interval between shown diets (p<0.05). The color of the asterisk signifies which diet has higher survivorship for that interval. (TIF) [file pone.0240132.s003.tif]

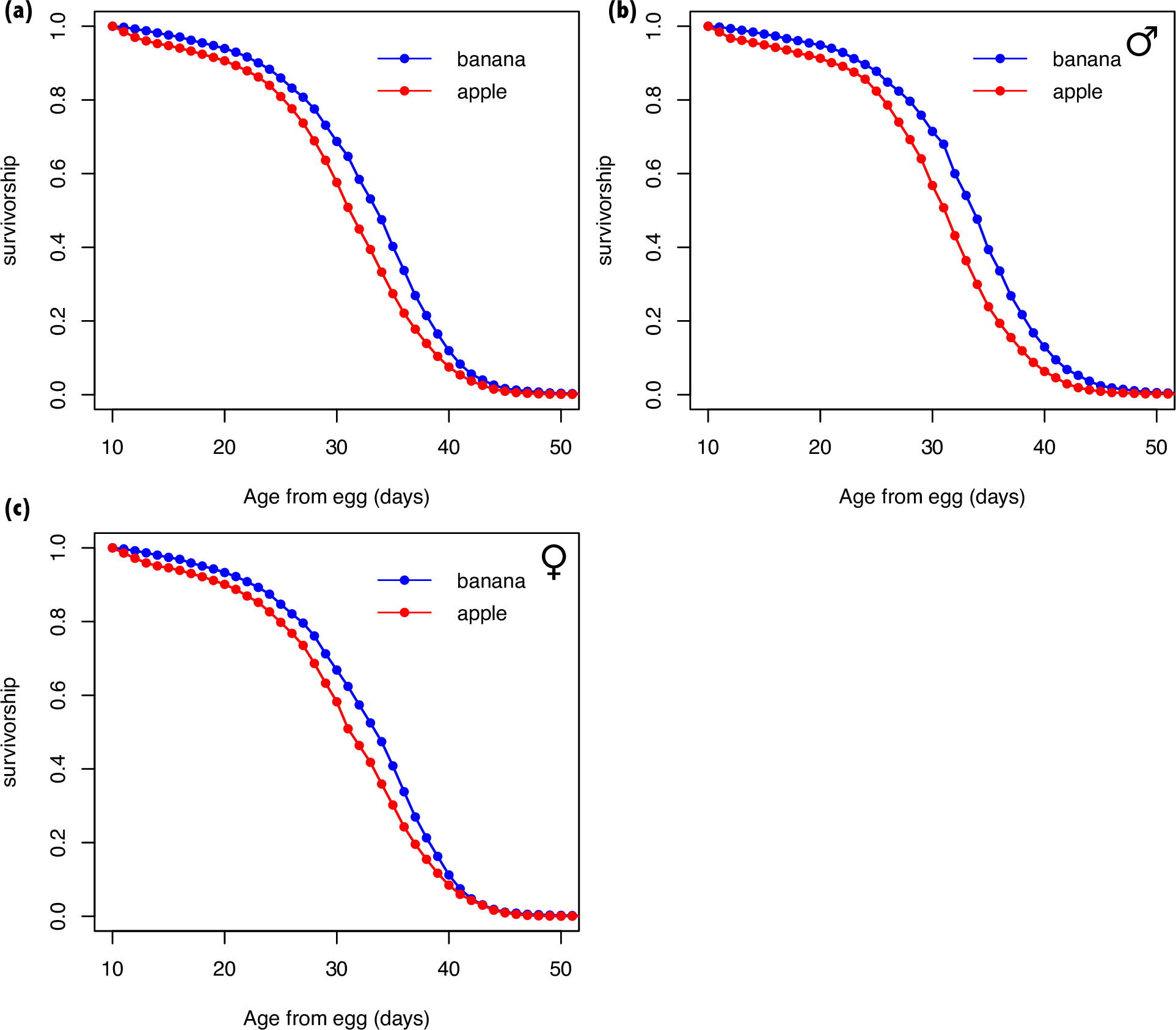

Supplement: S4 Fig — (a) sexes pooled. (b) male flies. (c) female flies. Data are pooled across replicates. (TIF) [file pone.0240132.s004.tif]

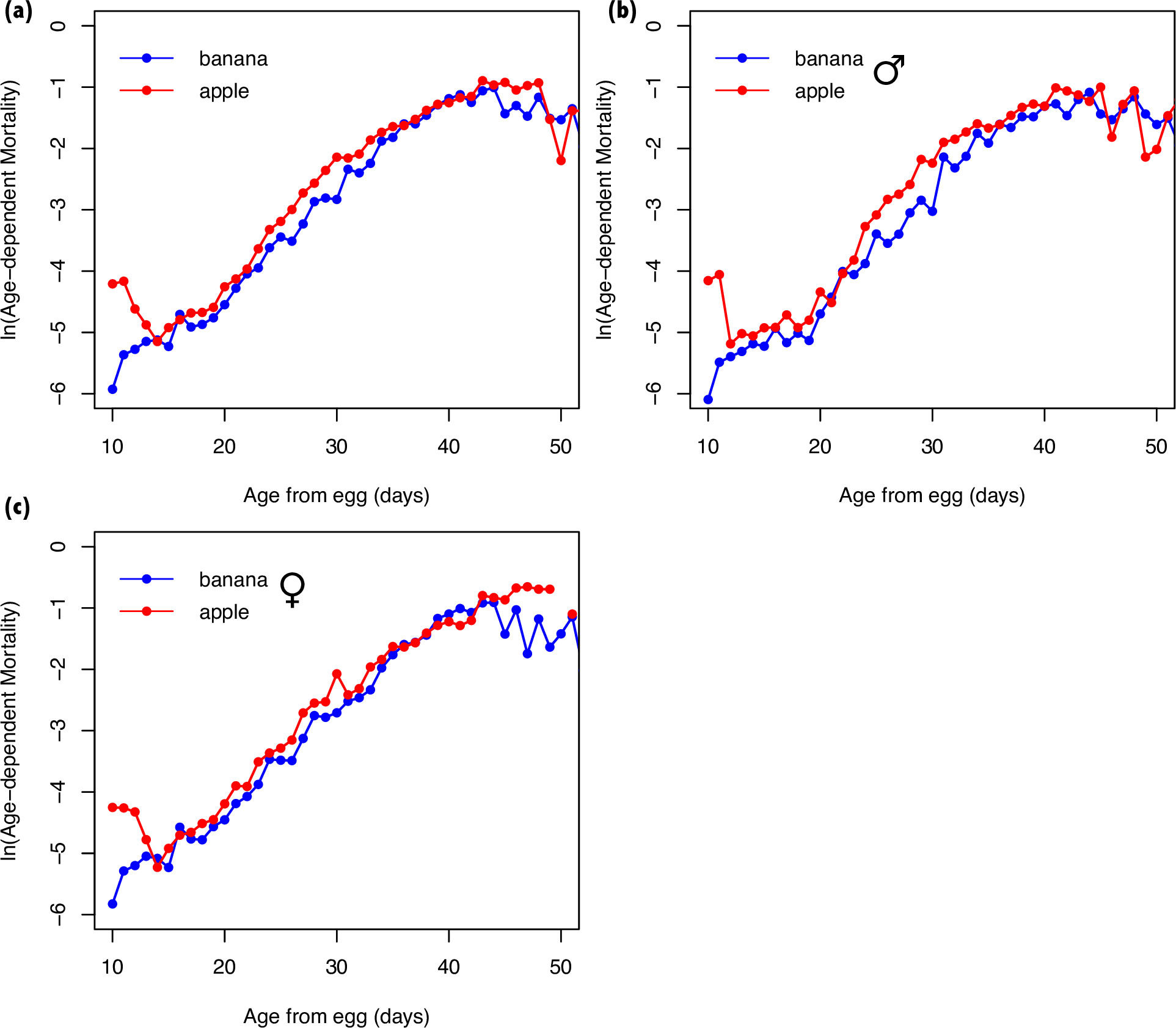

Supplement: S5 Fig — (a) sexes pooled. (b) male flies. (c) female flies. Data are pooled across replicates. (TIF) [file pone.0240132.s005.tif]

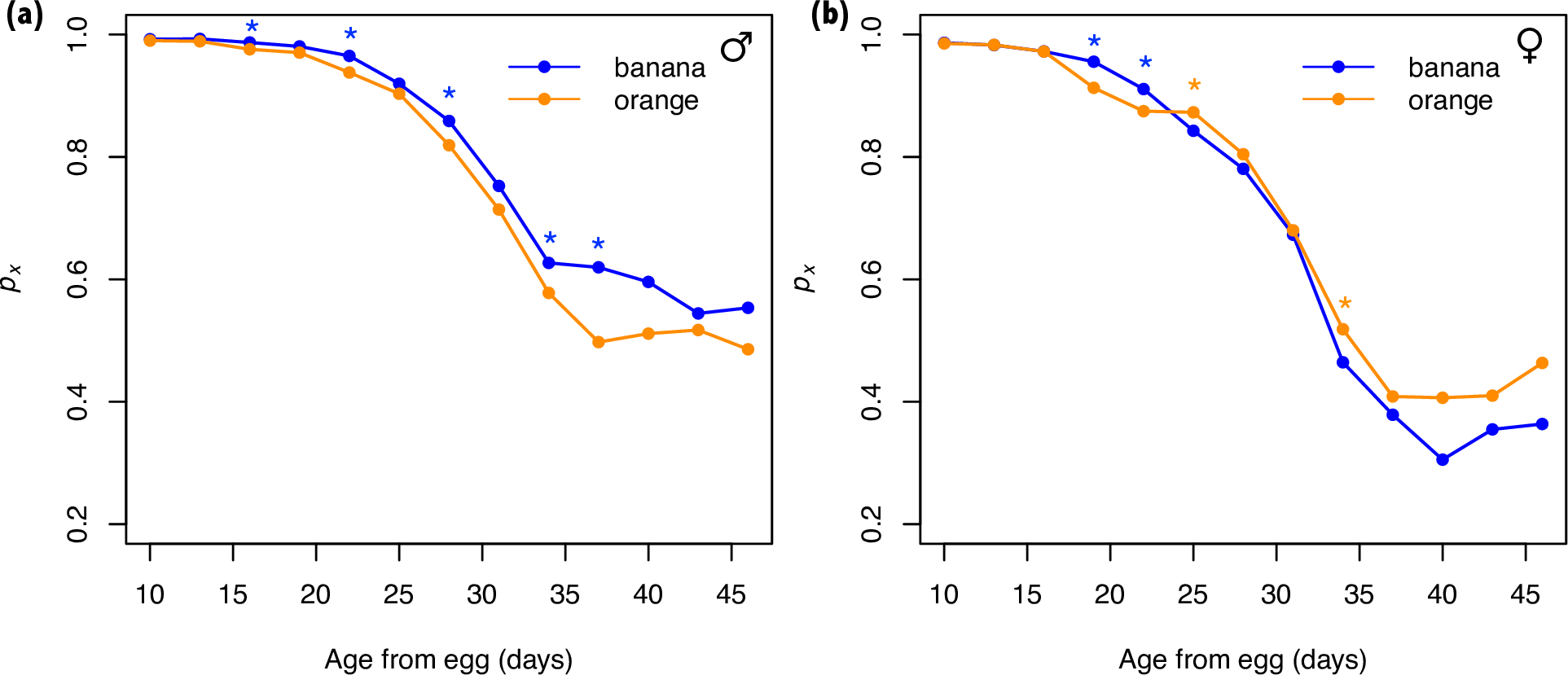

Supplement: S6 Fig — (a) males and (b) females. Points represent px pooled across replicates and pooled across three days. *denotes significance for the particular interval between shown diets (p<0.05). The color of the asterisk signifies which diet has higher survivorship for that interval. (TIF) [file pone.0240132.s006.tif]

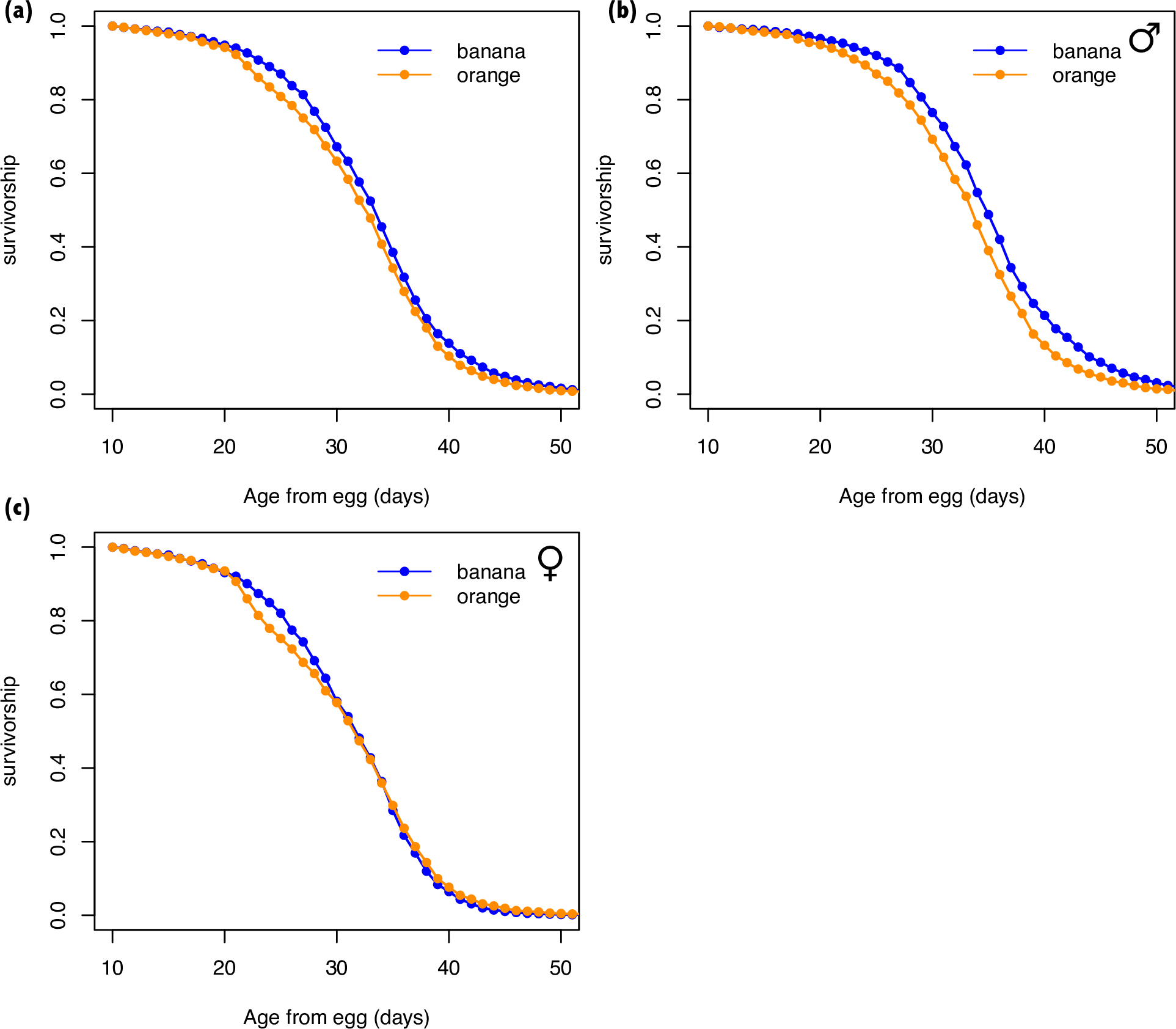

Supplement: S7 Fig — (a) sexes pooled. (b) male flies. (c) female flies. Data is pooled across replicates. (TIF) [file pone.0240132.s007.tif]

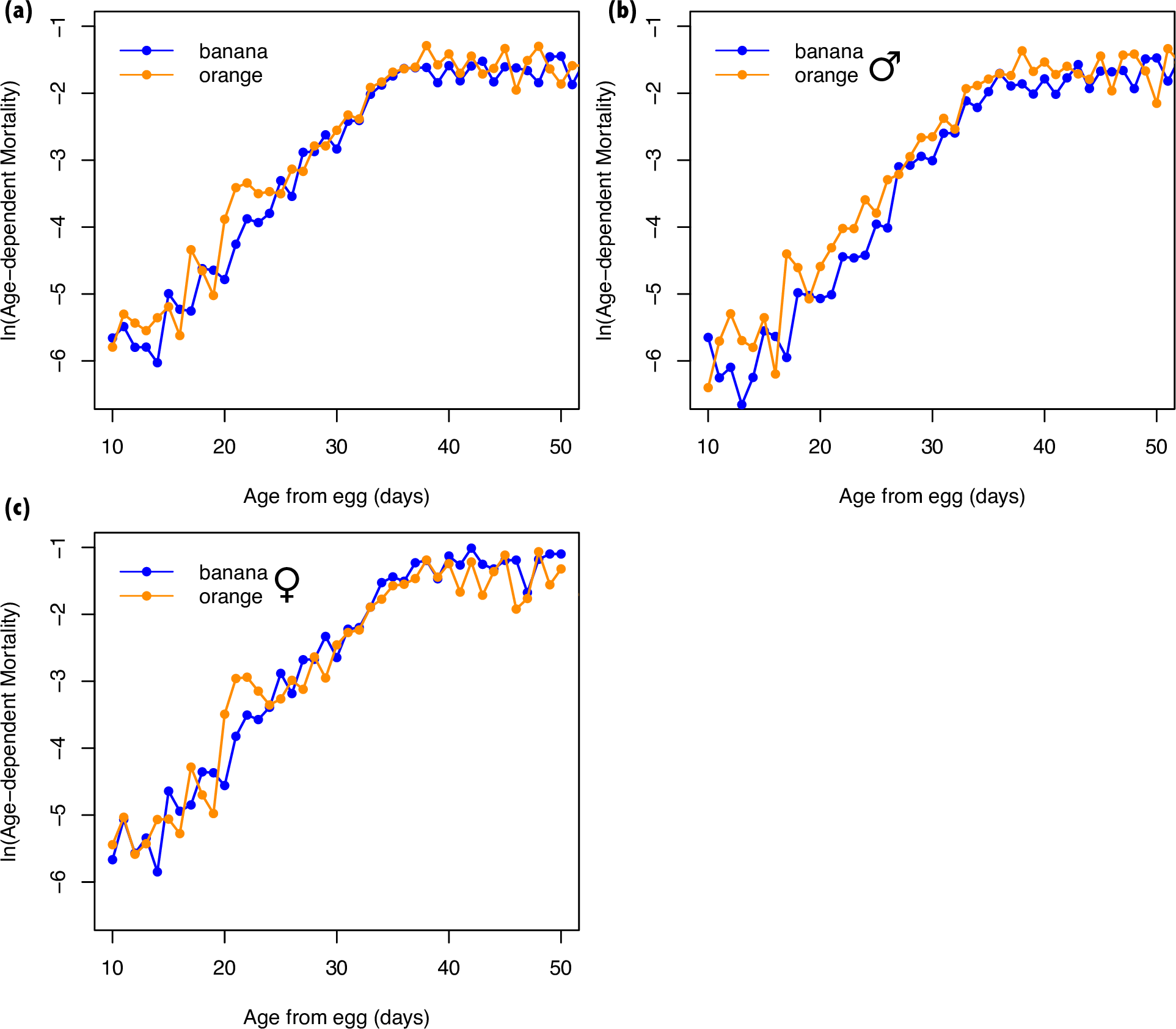

Supplement: S8 Fig — (a) sexes pooled. (b) male flies. (c) female flies. Data are pooled across replicates. (TIF) [file pone.0240132.s008.tif]

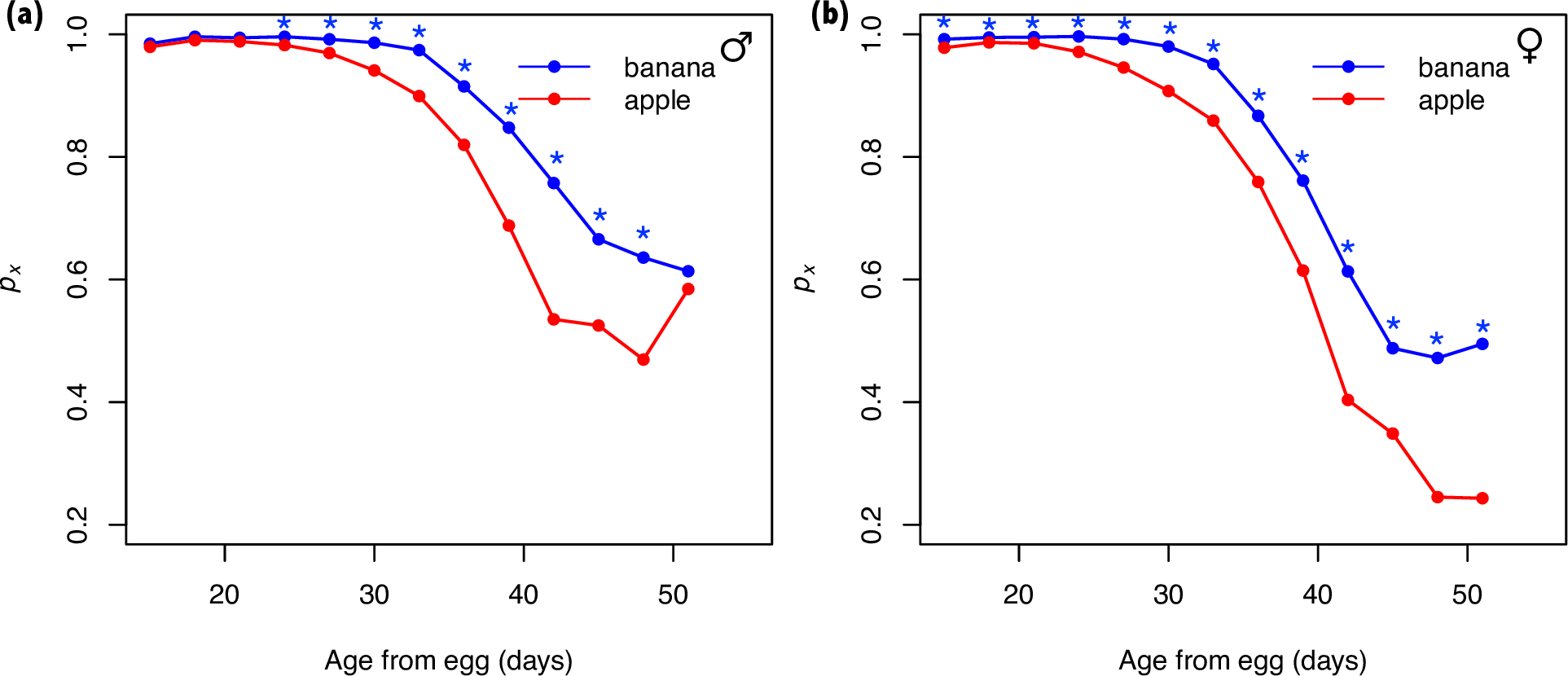

Supplement: S9 Fig — (a) males and (b) females. Points represent px pooled across replicates and pooled across three days. *denotes significance for the particular interval between shown diets (p<0.05). The color of the asterisk signifies which diet has higher survivorship for that interval. (TIF) [file pone.0240132.s009.tif]

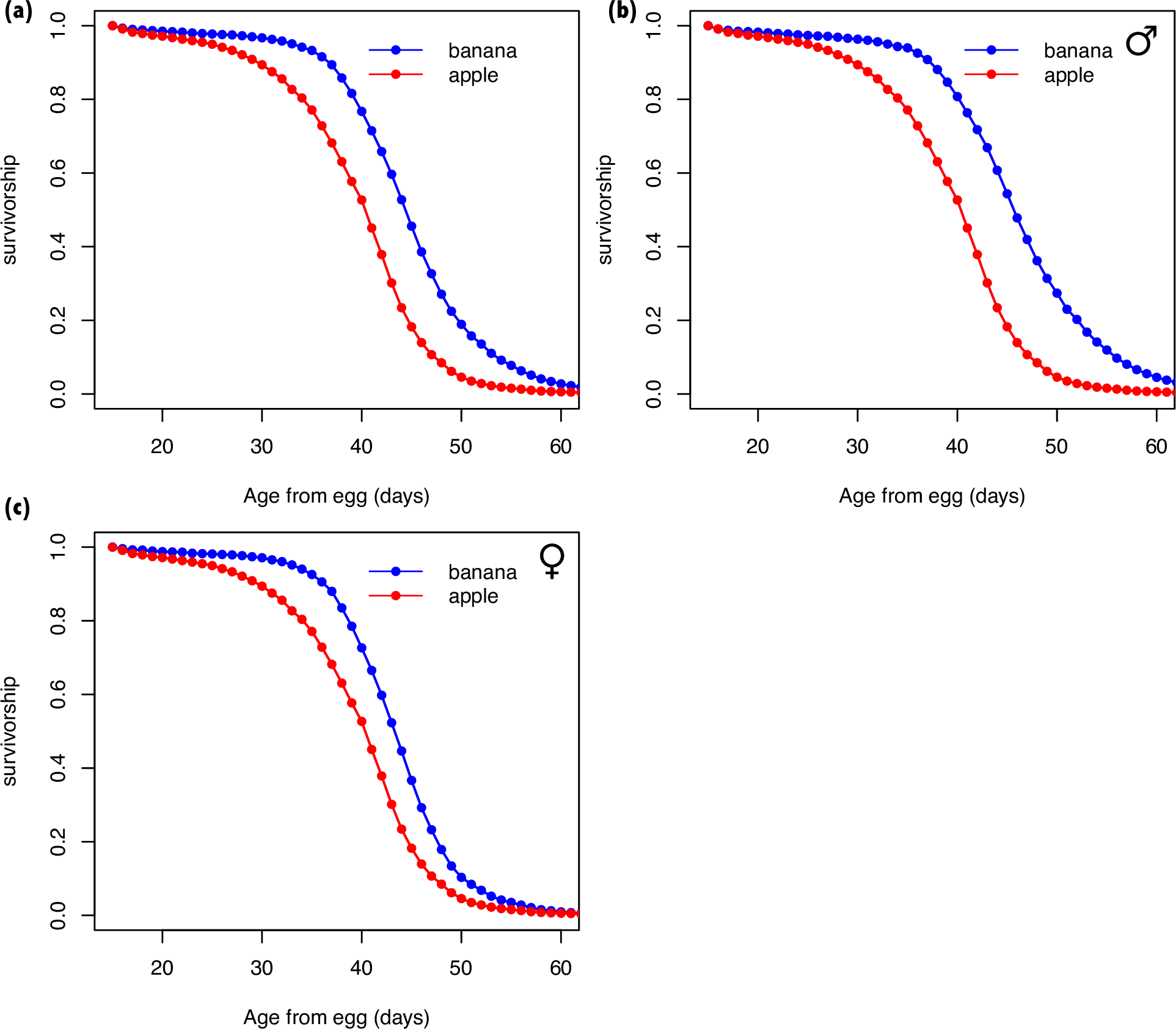

Supplement: S10 Fig — (a) sexes pooled. (b) male flies. (c) female flies. Data are pooled across replicates. (TIF) [file pone.0240132.s010.tif]

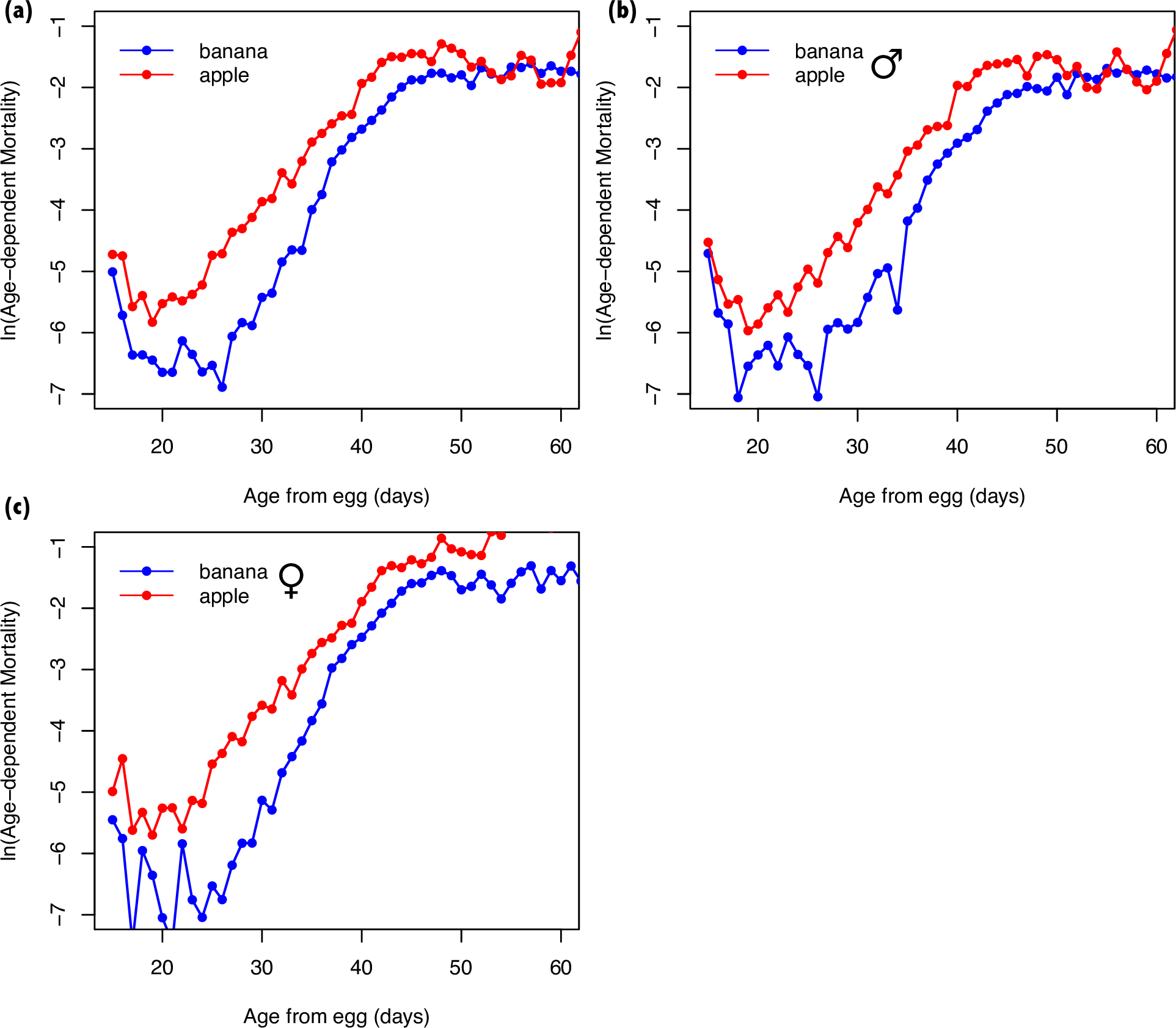

Supplement: S11 Fig — (a) sexes pooled. (b) male flies. (c) female flies. Data are pooled across replicates. (TIF) [file pone.0240132.s011.tif]
