# Supplementary material for: Hamiltonian patterns of age-dependent adaptation to novel environments
Source: PLoS One. 2020 Oct 2;15(10):e0240132. doi: 10.1371/journal.pone.0240132 (PMC7531798; doi:10.1371/journal.pone.0240132)
Supplement: S1 Table — (DOCX) [file pone.0240132.s012.docx]

**Table S1 Basic nutritional facets for three different diets used in the diet manipulation experiments.**

|  | Banana (1L) | Orange (1L) | Apple (1L) |
| --- | --- | --- | --- |
| Total Fat (g) | 1.2 | 0.9 | 0.7 |
| Sugar (g) | 37.2 | 32.2 | 11.1 |
| Total Carbs (g) | 90.36 | 76.4 | 26.5 |
| Protein (g) | 21.2 | 20.9 | 19.7 |
| Calories (Kcal) | 450 | 395.6 | 197.66 |
| Calculations from the fruit, yeast, and syrups | | | |
